# The pattern of *rpoB* gene mutation of *Mycobacterium tuberculosis* and predictors of rifampicin resistance detected by GeneXpert MTB/RIF assay in Tanzania

**DOI:** 10.1371/journal.pone.0296563

**Published:** 2024-08-26

**Authors:** Peter Richard Torokaa, Mtebe V. Majigo, Heledy Kileo, Loveness Urio, Mariam R. Mbwana, Mariam C. Monah, Sephord Saul Ntibabara, Jasper Kimambo, Paschal Seleman, Collins Franklin, Robert Balama, Riziki M. Kisonga, Agricola Joachim

**Affiliations:** 1 Muhimbili University of Health and Allied Sciences, School of Public Health and Social Sciences, Dar es Salaam, Tanzania; 2 Tanzania Field Epidemiology and Laboratory Training Program, Dar es Salaam, Tanzania; 3 Muhimbili University of Health and Allied Sciences, School of Diagnostic Medicine, Dar es Salaam, Tanzania; 4 Ministry of Health, National TB and Leprosy Programme, Dodoma, Tanzania; The University of Georgia, UNITED STATES OF AMERICA

## Abstract

**Introduction:**

Antimicrobial resistance in *Mycobacterium tuberculosis (*MTB*) poses a* significant challenge to tuberculosis (TB) management worldwide. Rifampicin resistance (RR) has been associated with the *rpoB* gene mutation. No study was conducted in Tanzania to determine the commonest mutation. The inconsistent findings from various studies support the need to determine whether reported mutation patterns are applicable in our setting. We determined the frequency of *rpoB* gene mutation and factors associated with RR, which were detected using GeneXpert MTB/RIF assay.

**Methods:**

We conducted a retrospective cross-sectional study involving data from the National Tuberculosis and Leprosy Program database from 2020 to 2022 for cases investigated using GeneXpert MTB/RIF assay. Descriptive analysis was performed to determine the frequency of categorical variables. The chi-square test and logistic regression analysis assessed the relationship between the independent variables and outcome. The 95% confidence interval and a significance level of p<0.05 were used to assess the strength of association.

**Results:**

A total of 56,004 participants had a status of MTB and RR, where 38,705/56,004 (69.11%) were males. Probe E mutation (codon 529–533), 89/219 (40.64%) was predominant. Human immunodeficiency virus (HIV)-positive patients had a higher gene mutation, 134/10601 (1.26%) than HIV-negative, 306/45016 (0.68%) (p<0.001). Patients with both pulmonary and extra-pulmonary TB had about four times greater odds of developing rifampicin resistance (AOR 3.88, 95%CI: 1.80–8.32). RR was nearly nine times higher in previously treated patients than new patients (AOR 8.66, 95% CI: 6.97–10.76). HIV-positive individuals had nearly twice the odds of developing RR than HIV-negative individuals (AOR 1.91, 95%CI: 1.51–2.42).

**Conclusion:**

The rate of RR was lower compared to other studies in Tanzania, with probe E mutations the most prevalent. Patients with disseminated TB, HIV co-infection and those with prior exposure to anti-TB had more risk of RR. The findings highlight the need to strengthen surveillance of multidrug-resistant TB among high risk patients.

## Introduction

Multidrug-resistant *Mycobacterium tuberculosis* (MDR-TB) is the biggest challenge facing Tuberculosis (TB) care and treatment worldwide [1]. MDR-TB is a major contributor to global antimicrobial resistance (AMR) and continues to pose a public health threat [1–3]. MDR-TB is considered when there is resistance to isoniazid and rifampicin, with or without resistance to other first-line drugs. The main problem is rifampicin resistance, the most potent first-line treatment [1]. Patients with rifampicin-resistant TB or MDR-TB require a second-line treatment regimen [4]. Globally, the annual estimated number of MDR-TB or rifampicin-resistant TB cases was steady between 2015 and 2020; however, it increased in 2021. In 2021, it was estimated to be 450,000 events, a 3.1% increase from 437,000 in 2020 [1].

The World Health Organization (WHO) developed a strategy to end TB-related deaths, illnesses, and suffering by 2035 [5]. It recommends accelerating the identification and enhancing treatment for MDR-TB. Apart from access to diagnosis, adequate infection control must also be implemented in the settings where patients are treated [4]. To diagnose MDR-TB, WHO recommends more specific and sensitive assays like real-time polymerase chain reaction (RT-PCR), DNA microarray, and loop-mediated isothermal amplification (LAMP). These technologies determine the mutations in the genes to identify drug resistance. In some populations of HIV-infected presumptive TB patients, the lateral flow lipoarabinomannan assay (LF-LAM) test is advised to aid in the diagnosis of TB [6].

The invention of the Xpert® MTB/RIF Assay (Cepheid, USA), which simultaneously detects the presence of *Mycobacterium tuberculosis* (MTB) and rifampicin resistance, has transformed the diagnosis of TB. Rifampicin inhibits DNA-directed RNA synthesis of MTB proteins by binding to the beta subunit of the bacterial DNA-dependent RNA polymerase *(rpoB)* enzyme. Rifampicin resistance has been associated with the *rpoB* gene mutation. Almost 96.1% of rifampicin-resistant MTB strains have *rpoB* mutations [7]. The rifampicin resistance is the proxy sign of the MDR-TB [8,9]. GeneXpert MTB/RIF assay is a cartridge-based, automated hemi-nested real-time PCR system that utilizes five overlapping probes named Probe A (codons 507–511), Probe B (codons 511–518), Probe C (codons 518–523), Probe D (codons 523–529) and Probe E (codons 529–533) [10] which are rifampicin resistance determining region. The device can detect MTB and its resistance to rifampicin directly from the patient’s sputum within two hours [11].

Studies in Africa revealed that the commonest mutation of *rpoB* occurs at probes E and D [12,13]. However, the literature shows different pattern distributions of the *rpoB* gene mutation of MTB associated with drug resistance [14–16]. There is a need to determine whether the findings in other studies apply to our setting due to the varied findings obtained in various geographical areas. No other study has been conducted in Tanzania to reveal the patterns of *rpoB* mutation. Due to efforts to increase access to GeneXpert MTB/RIF assay for diagnosis in the country, there is also a need to determine the factors associated with rifampicin-resistant TB.

## Methods

### Study design and setting

We conducted a retrospective cross-sectional study involving the Tanzania National Tuberculosis and Leprosy Program (NTLP) data collected from January 2020 through December 2022. The study covered Presumptive TB cases notified to NTLP from 26 regions of Tanzania’s mainland. The NTLP is tasked to prevent tuberculosis and leprosy as serious public health concerns in Tanzania, and it was launched in 1977 as a single combined programme for the two diseases.

### Data collection

We extracted demographic and clinical data from electronic TB and leprosy register (eTL) and laboratory results from GxAlert databases on 10^th^ October 2023 and then exported them to Microsoft Excel. The demographic data from the eTL register and laboratory results data were linked between these two databases. The identifiers used to link the two databases were patient name, health facilities, district, and year. The variables of interest from the two databases were demographic characteristics (age, gender, residence), clinical characteristics (TB treatment history, type of TB, bacillary load, HIV status), and GeneXpert MTB/RIF assay results.

### Detection of RIF resistance

RIF resistance and *rpoB* mutations are identified by comparing the Cycle threshold (C_T_) values of the initial and final *M*. *tuberculosis*-specific molecular beacons (ΔC_T_ Max). A ΔC_T_ Max of >3.5 cycles indicate RIF resistance, whereas a value of ≤3.5 cycles indicate susceptibility. If the initial probe reveals a C_T_ value of more than 34.5 cycles and the last probe yields a C_T_ of more than 38, the sample is deemed RIF indeterminate [17–20]. In this study, *rpoB* mutations that induce a quantifiable ΔC_T_ Max of >3.5 cycles and prevent probe hybridization are referred to as "missing probe," whereas mutations that allow for partial probe hybridization are referred to as "no missing probe” [21].

### Data extraction and Interpretation

**Fig 1:**
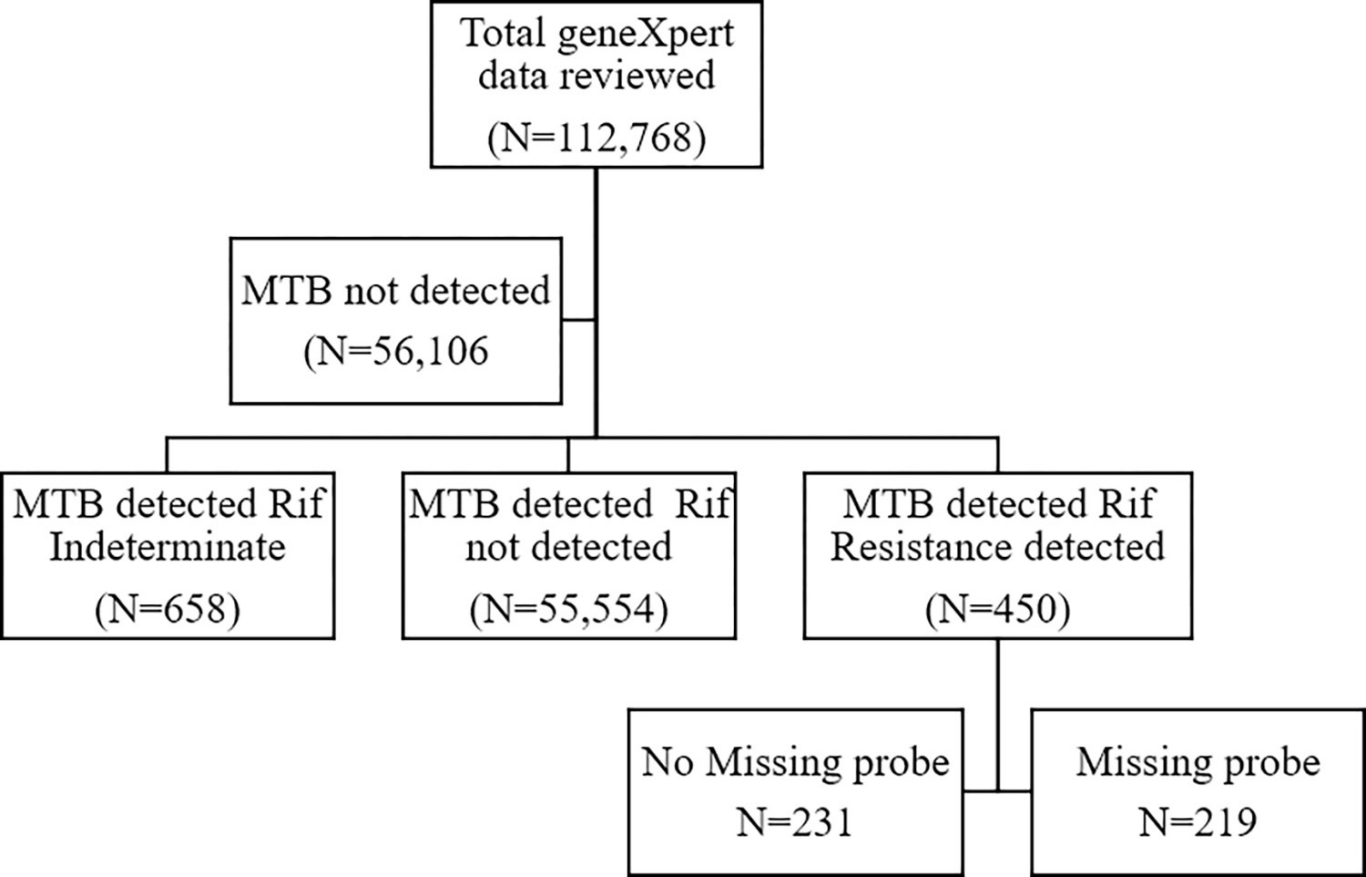
Flow chart of participants’ analysis.

We extracted data for 112,768 non-repeating patients tested for TB using GeneXpert MTB/RIF assay. Of all, 56,662 (50.2%) had laboratory-confirmed TB. Rifampicin resistance was identified in 450/56,662 (0.8%) patients with MTB (S1 File). The specific gene mutations in the *rpoB* gene of MTB were identified in the form of the missing probes, which indicated a specific mutation occurs at a specific codon in the *rpoB* gene named Probe A (codons 507–511), Probe B (codons 511–518), Probe C (codons 518–523), Probe D (codons 523–529), and Probe E (codons 529–533). Missing probes (specific *rpoB* mutation detected) were found in 219/ 450 (48.7%) samples with Rifampicin-resistant MTB, and no missing probes (no specific *rpoB* mutation detected) in 231/450 (51.3%) with rifampicin-resistant. RIF Indeterminate were observed in 658/56,662 (1.16%) of patients with MTB and were excluded in further analysis (Fig 1). The GeneXpert MTB/RIF assay results were reported as MTB (M tuberculosis) DETECTED; Rif (rifampicin) resistance DETECTED, MTB DETECTED; RIF resistance NOT DETECTED, MTB detected; RIF resistance INDETERMINATE, MTB NOT DETECTED, INVALID (the presence or absence of MTB cannot be determined), ERROR (the presence or absence of MTB cannot be determined), NO RESULT the presence or absence of MTB cannot be determined [22].

### Data analysis

We presented the descriptive analysis with frequency distributions (%) for categorical variables, and a mean was a measure of central tendency for age. Logistic regression analyses (bivariate and multivariate) were performed to assess the relationship between demographic and clinical characteristics as independent variables and the rifampicin resistance as an outcome variable. Factors with a p-value of 0.20 in the bivariate analyses were included in the multivariable model using forward selection. The 95% confidence interval (CI) was presented, and a significance level of p <0.05 was used.

### Ethics statement

Ethical approval was obtained from the Senate Research and Publications Committee of Muhimbili University of Health and Allied Sciences, Ref. No. DA.282/298/01L/629. Approval to use the national TB program data was obtained from the NTLP Program Manager in the Ministry of Health (MoH). The data was routinely collected by health facilities providing TB care and treatment services in which ethical issues are strongly advocated.

## Results

### Demographic and clinical characteristics of study participants

**Fig 2:**
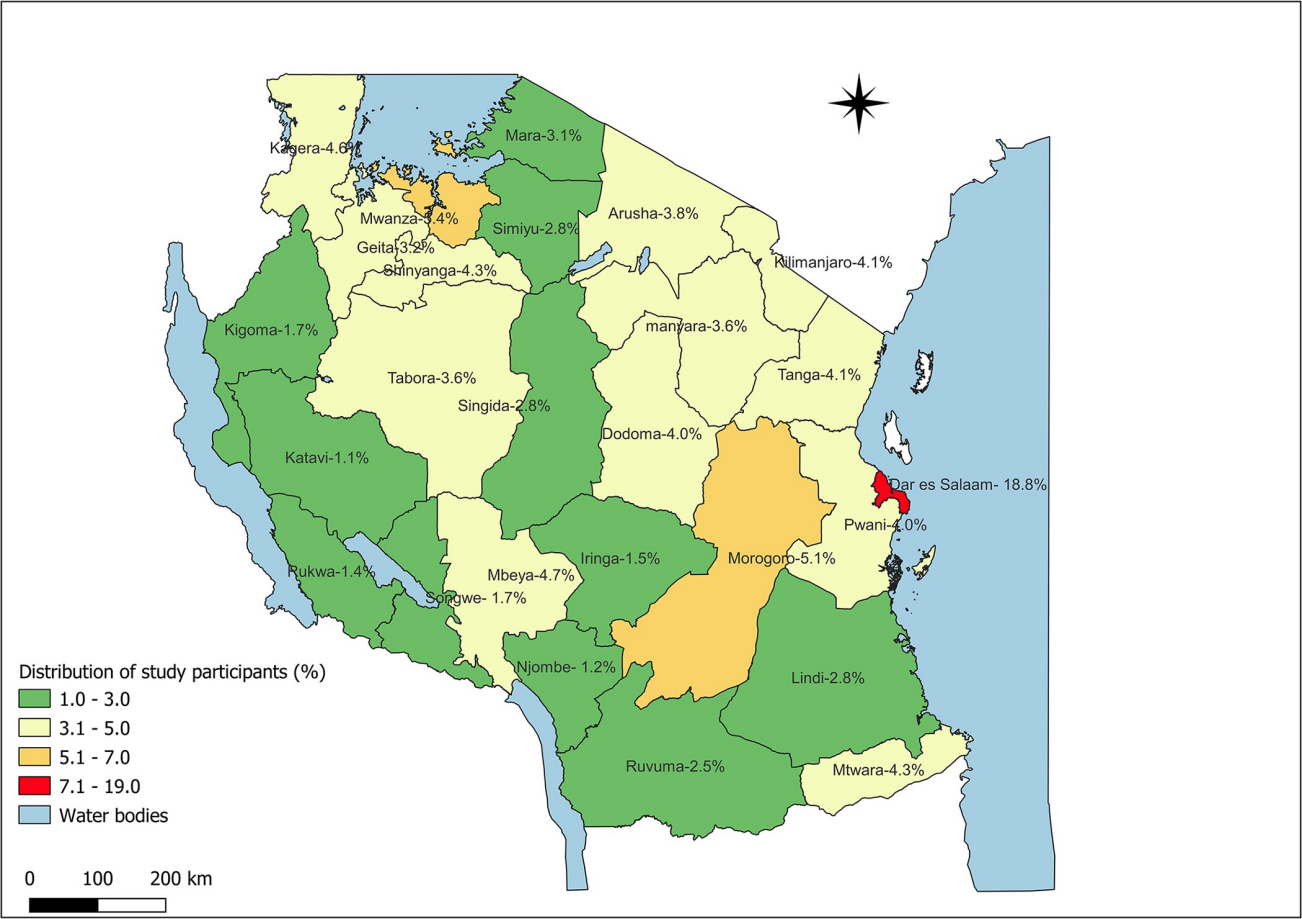
Map showing the proportion distribution of study participants per region. The map shows 26 Tanzania mainland administrative regions. (This map was adapted from the Tanzania NBS): (https://www.nbs.go.tz/nbs/takwimu/references/Licence-Agreement-NBS.pdf).

A total of 56,004 participants had clear status of MTB and rifampicin resistance (S1 File) where majority 38,705/56,004 (69.11%) were males. The age group 35–44 years accounted for the largest cases, 13,512/56,004 (24.13%). Most participants, 55,192/56,004 (98.55%), had pulmonary TB. Participants with new TB were 53,818/56,004 (96.1%), and 45,016/56,004 (80.38%) were HIV-negative (Table 1). The Dar es Salaam region contributed 10,524/56,004 (18.79%) cases, which is higher than in any other region. The lowest number of cases were reported from the Katavi region. The regional distribution of cases is shown in Fig 2.

**Table 1:** Participants’ demographic and clinical characteristics (N = 56,004).

Variable	Frequency	Percent (%)
**Age Group (years)**		
<15	1765	3.15
15–24	6441	11.5
25–34	12637	22.56
35–44	13512	24.13
45–54	10460	18.68
>55	11189	19.98
Mean age (SD)	41(16.4)	
**Gender**		
Male	38705	69.11
Female	17299	30.89
**TB Type**		
Pulmonary	55192	98.55
Extra pulmonary	550	0.98
Pulmonary and extra pulmonary	262	0.47
**Treatment History**		
New patient	53818	96.1
Previous treated	2186	3.9
**HIV status**		
Positive	10601	18.93
Negative	45016	80.38
Unknown	387	0.69

### Pattern of specific *rpoB* gene mutations in MTB

Specific mutations (missing probe) were detected in 219/450 (48.7%) rifampicin-resistant TB. The most prevalent mutation, 89/219 (40.64%), occurred at Probe E, followed by Probe D, 44/219 (20.09%), and the least mutation, 10/219 (4.56%), occurred at Probe C. We found a mutation combination of Probe A and B, 5/219 (2.28%), Probe A and D, 2/219 (0.91%), Probe A and E 1/219 (0.46%), and Probe B and E 1/219 (0.46%). One patient had a triple mutation combination at Probe A, D, and E (0.46%) (Table 2).

**Table 2:** Frequency and distribution of specific *rpoB* gene mutations (N = 219).

Probes	#Codons	Frequency	Percent (%)
Probe A	507–511	21	9.59
Probe B	511–518	39	17.81
Probe C	518–523	10	4.57
Probe D	523–529	44	20.09
Probe E	529–533	89	40.64
Probe A+B		5	2.28
Probe A+D		2	0.91
Probe A+E		1	0.46
Probe B+E		1	0.46
Probe A+D+E		1	0.46

### The proportion of participants with no missing probe

Specific *rpoB* mutation were not detected (no missing probe) in 231/450 (51.3%) participants with Rifampicin-resistance. We found that no missing probes were significantly higher in new TB patients, 189/334 (56.59%) (p = 0.003). Patients with a very low TB bacillary load, 199/263 (75.67%), (p<0.001) had a higher number of no missing probes. There was no statistically significant difference of no missing probes for other variables (Table 3).

**Table 3:** Demographic and clinical characteristics of the number of no-missing probe study participants (N = 231).

Variable	Total Rifampicin Resistant	# No missing probe	Proportion	^1^P value
**Age Group**				
<15	6	3	50.00	
15–59	376	185	49.20	
>59	68	43	63.24	0.103
**Gender**				
Male	312	168	53.85	
Female	138	63	45.65	0.133
**TB Type**				
Pulmonary	438	224	51.14	
Extra pulmonary	5	2	40.00	
Pulmonary and extra pulmonary	7	5	71.43	0.599
**Treatment History**				
New patient	334	189	56.59	
Previous treated	116	42	36.21	0.003
**HIV status**				
Positive	134	59	44.03	
Negative	306	167	54.58	0.125
**Bacillary Load**				
High	61	10	16.39	
Medium	66	5	7.58	
Low	60	17	28.33	
Very low	263	199	75.67	<0.001

^**1**^p- value for chi-squared test.

### Rifampicin resistance and participant characteristics

The proportion of *rpoB* gene mutation was higher in 15-59-year-old patients, 383/45,782 (0.84%) (p = 0.047). High frequency of mutations was observed in patients with pulmonary and extra-pulmonary TB patients (7/262, 2.67%) (p = 0.003). The previously treated patients had a high mutation rate, 116/2186 (5.31%) (p<0.001). HIV-positive TB patients had a higher gene mutation, 134/10601 (1.26%) (p<0.001). Other variables like gender and point of health service showed no significant difference in gene mutation (Table 4).

**Table 4:** Distribution of rifampicin resistance and participants’ characteristics (N = 56,004).

Variable	Total participants	# Total RR	Proportion	P value
**Age Group**				
<15	1765	6	0.34	
15–59	45782	383	0.84	
>59	8457	61	0.72	0.047
**Gender**				
Male	38705	312	0.81	
Female	17299	138	0.80	0.918
**TB Type**				
Pulmonary	55192	438	0.79	
Extra pulmonary	550	5	0.91	
Pulmonary and extra pulmonary	262	7	2.67	0.003
**Treatment History**				
New patient	53818	334	0.62	
Previous treated	2186	116	5.31	<0.001
**HIV status** **				
Positive	10601	134	1.26	
Negative	45016	306	0.68	<0.001
**Referral type**				
Community	46897	364	0.78	
Health facility	6167	60	0.97	
Others	2940	26	0.88	0.234

** The frequency for HIV status is less due to unknown status.

### Predictors of rifampicin resistance

We found that patients with both pulmonary and extra-pulmonary TB had about four times greater odds of developing rifampicin resistance (AOR 3.88, 95%CI: 1.80–8.32). The likelihood of rifampicin resistance was nearly nine times higher in previously treated patients compared to new patients (AOR 8.66, 95%CI: 6.97–10.76). HIV-positive individuals had nearly twice the odds of developing rifampicin resistance than HIV-negative individuals (AOR 1.91, 95%CI: 1.51–2.42) (Table 5).

**Table 5:** Multivariate logistic regression analysis for the predictors of Rifampicin resistance.

Characteristic	Rif. resistance n (%)	COR (95% CI)	P-value	AOR (95% CI)	P-value
**Age Group**					
<15	6 (0.34)	Ref		Ref	
15–59	383 (0.84)	2.47 (1.10–5.55)	0.028	2.07 (0.92–4.67)	0.078
>59	61 (0.72)	2.13 (0.92–4.93)	0.078	1.95 (0.84–4.54)	0.119
**TB Type**					
Pulmonary	438 (0.79)	Ref		Ref	
Extra pulmonary	5 (0.91)	1.15 (0.47–2.78)	0.762	1.19 (0.49–2.92)	0.694
Pulmonary and extra pulmonary	7 (2.67)	3.43 (1.61–7.31)	0.001	**3.88 (1.80–8.32)**	**0.001**
**Treatment History**					
New patient	334 (0.62)	Ref		Ref	
Previous treated	116 (5.31)	8.97 (7.23–11.13)	<0.001	**8.66 (6.97–10.76)**	**<0.001**
**HIV status**					
Negative	306 (1.26)	Ref		Ref	
Positive	134 (0.68)	1.87 (1.53–2.29)	<0.001	**1.82 (1.44–2.30)**	**<0.001**
**Referral type**					
Community	364 (0.78)	Ref		Ref	
Health facility	60 (0. 97)	1.25 (0.95–1.65)	0.104	0.83 (0.61–1.14)	0.250
Others	26 (0.88)	1.14 (0.76–1.70)	0519	0.99 (0.66–1.48)	0.969

## Discussion

Our study has revealed that the rate of rifampicin resistance in patients with MTB is nearly 1%. However, a specific *rpoB* mutation was detected in around 49% of samples with rifampicin-resistant MTB. The most prevalent mutation occurred at probe E among the five probes and contributed to around 41 out of 100 mutations detected. The proportion of RR-TB was significantly higher among previously treated TB patients. The current study found that patients with pulmonary and extra-pulmonary TB, previously treated, HIV-positive individuals, had greater odds of developing rifampicin resistance compared to their counterparts.

The rate of rifampicin resistance was found to be lower in our study than in earlier studies done in Tanzania ranging from 8.3 to 12.7% in these studies [23–25]. The difference between previous studies findings and ours could be explained by the use of a large sample size, which showed a lower rate. Our study findings revealed a lower rate compared to the past seven years, which could be explained by an improved and well-functioning TB control program that includes decentralization of DR-TB patient care services that reduces lost to follow-up [26].

The current study found that the most prevalent mutation in *rpoB* gene mutation in MTB was at probe E codon (529–533) (40.64%), followed by Probe D codon (523–529) 20.09% which was similar to studies done in Nigeria, Uganda, Pakistan, Addis Ababa Ethiopia, and Bangladesh [16,21,27–29]. The similarities suggests that a significant number of low-income individuals migrating for shelters may be responsible for the spread of mutant strains within the community [30]. Our findings differed from the study done in Northeast India and Enugu, South Eastern Nigeria, which revealed that the most prevalent mutations were detected at probes A and D, respectively [12,13]. The frequency and patterns of *rpoB* mutations can vary geographically, with certain mutations more prevalent in specific regions or variations in the *M*. *tuberculosis* lineage [31]. According to the results of our study and previous studies, probe E is linked to the most common probe mutation in the *rpoB* gene mutation [16,21,27–29].

A high proportion (51.3%) of no missed probe of the *rpoB* gene was detected in this study. Our finding is higher than the previously report by Alemu A, et al., who found 6% mutations conferring RR TB without any missed probe types [21]. Furthermore, study by Akalu GT, et al., reported a significant proportion (12.5%) of RRTB patients were found without unidentified missed probe detected outside of the RRDR [32]. High rates of no missing probes identified outside of the RRDR have been linked with the changes in threshold cycles and low DNA amount, where in our study the CT value was >3.5 while in other studies were of > 4.0 [21,32]. The differences could also be due to an early stage of TB diagnosis, which may have a low bacillary load. No missing probe may also be triggered by various probes having varying target hybridization dynamics, which might have a higher impact after extended PCR cycles [33]. Patients with low TB bacillary load also had more no-missing probes, possibly due to insufficient amplification of particular probe sequences and leading to attachment failure resulting to false-positive rifampicin resistance [34]. The very low bacillary load on GeneXpert MTB/RIF assay testing was shown to be substantially linked to false rifampicin resistance during the initial GeneXpert MTB/RIF assay [33]. The samples with very low bacillary load were not retested; hence, the rate of false positives for rifampicin resistance could not be determined. Furthermore, a study using sequencing has also reported a significant proportion (1.94%) of mutations identified outside the RRDR such as at P280L, E521K, and D595Y which may contribute to rifampicin resistance [35]. Thus, further investigation including sequencing is needed to ascertain the high rates of RR detected without missed probe in this study.

The rifampicin resistance was found more in patients with both pulmonary and extra-pulmonary TB than in pulmonary or extra-pulmonary alone. In addition, patients with both pulmonary and extra-pulmonary TB had almost four times greater odds of developing rifampicin resistance. Our findings were comparable to the study done in the Debre Markos Referral Hospital Ethiopia [36]. We also found that previously treated patients had significantly higher rifampicin resistance than new patients. The likelihood of rifampicin resistance was nearly nine times higher in previously treated patients than in new patients, similar to the study done in Nepal [37]. Our findings support that MTB increases the ability to develop resistance when exposed to anti-TB drugs, especially in patients with poor adherence to treatment [38]. In our study, the odds of developing rifampicin resistance in previously treated patients were higher than in Somalia and other East Gojjam zone northwest Ethiopia studies, which revealed four and six times, respectively [39,40]. The use of GeneXpert MTB/RIF assay in testing previously TB treated patients helps to identify drug-resistant strains more quickly, allowing for timely adjustments to treatment plans [11]. However, the assay should not be used for monitoring patients during treatment since the assay detect DNA from both viable and non-viable bacilli.

HIV-positive TB patients had a higher gene mutation than HIV-negative. HIV-positive individuals had nearly twice the odds of developing rifampicin resistance than HIV-negative individuals; this was similar to the studies done in northwest Ethiopia [39,41]. Variations in TB/HIV co-infection have been reported in several studies, indicating challenges in diagnosis and treatment due to unusual clinical presentations and difficulties in diagnosis and treatment [42,43]. This may be due to differences in TB control strategies and approaches [41]. More rifampicin-resistant MTB in HIV patients might be caused by poor treatment adherence. Poor adherence to treatment is the primary factor contributing to drug resistance [37,42]. This highlights the need for more surveillance and community involvement.

The study had limitations, including missing variables and being unable to confirm the validity of high proportion of no missing probes for rifampicin resistance at low bacillary load. Furthermore, we were unable to confirm rifampicin resistance since phenotypic drug susceptibility testing or sequencing was not performed. However, the large sample size of participants from all Tanzania mainland regions made the findings representative and generalizable to all regions.

### Conclusion

The rate of rifampicin resistance in our study was lower compared to other studies done in Tanzania. The Probe E (codons 529–533)-related mutations were the most prevalent *rpoB* gene mutation. Patients with disseminated TB, HIV co-infection and those with prior exposure to anti-TB are associated with rifampicin resistance. The findings highlight the need to strengthen the surveillance of MDR-TB among patients identified with a higher risk of rifampicin resistance. We recommend further study be done using Xpert® MTB/RIF Ultra and rifampicin resistance-associated mutations and sequencing or phenotypic testing for no missing probes results.

## Supporting information

S1 File Distribution of detected MTB participants. (ZIP)
